# Cryogenic Transmission Electron Microscopy Nanostructural Study of Shed Microparticles

**DOI:** 10.1371/journal.pone.0083680

**Published:** 2013-12-26

**Authors:** Liron Issman, Benjamin Brenner, Yeshayahu Talmon, Anat Aharon

**Affiliations:** 1 Department of Chemical Engineering and The Russell Berrie Nanotechnology Institute (RBNI), Technion-Israel Institute of Technology, Haifa, Israel; 2 The Bruce Rappaport Faculty of Medicine, Technion-Israel Institute of Technology, Haifa, Israel; 3 Thrombosis and Hemostasis Unit, Department of Hematology, Rambam Health Care Campus, Haifa, Israel; University of Heidelberg Medical School, Germany

## Abstract

Microparticles (MPs) are sub-micron membrane vesicles (100–1000 nm) shed from normal and pathologic cells due to stimulation or apoptosis. MPs can be found in the peripheral blood circulation of healthy individuals, whereas elevated concentrations are found in pregnancy and in a variety of diseases. Also, MPs participate in physiological processes, e.g., coagulation, inflammation, and angiogenesis. Since their clinical properties are important, we have developed a new methodology based on nano-imaging that provides significant new data on MPs nanostructure, their composition and function. We are among the first to characterize by direct-imaging cryogenic transmitting electron microscopy (cryo-TEM) the near-to-native nanostructure of MP systems isolated from different cell types and stimulation procedures. We found that there are no major differences between the MP systems we have studied, as most particles were spherical, with diameters from 200 to 400 nm. However, each MP population is very heterogeneous, showing diverse morphologies. We investigated by cryo-TEM the effects of standard techniques used to isolate and store MPs, and found that either high-g centrifugation of MPs for isolation purposes, or slow freezing to –80°C for storage introduce morphological artifacts, which can influence MP nanostructure, and thus affect the efficiency of these particles as future diagnostic tools.

## Introduction

Microparticles (MPs) are sub-micron membrane vesicles (100–1000 nm in diameter) shed from normal and malignant cells due to stimulation or apoptosis. MPs can be found in the peripheral blood circulation of healthy individuals, whereas elevated concentrations are found in pathological conditions, including acute coronary disorders, peripheral arterial disease, systemic inflammatory diseases, diabetes, various types of cancer, and in pregnancy [1]. MPs have been documented to participate in processes of coagulation, inflammation, vascular reactivity, angiogenesis, metastasis, and the balance between cell proliferation, differentiation, cell survival and apoptosis [2]–[4]. This is possible because they carry cellular components from their cells of origin, including bioactive lipids, membrane-bound proteins, cytosolic proteins, viral contaminants, mRNA, and even organelles and DNA fragments [3]–[5]. These components can be transported to other cells via membrane fusion, or engulfment of MPs with the target cell membrane [6].

Most current conventional techniques, used to study microparticles, cannot analyse properly and fully MPs, or vesicles and liposome morphology [7], [8]. Even flow cytometry, which is the most common method to study MPs, offers a lower detection limit of only 300–500 nm [9], [10]. Consequently, only a fraction of MPs can be detected, and no morphological information about them can be acquired. Moreover, quantitative size information is obtained by comparing the scattering intensity of MPs with that of beads of known size. These measurements are not precise, as the scattering intensity depends not only on size, but also on shape, refractive index, and absorption of the analysed particles [11]. Flow cytometry analysis suffers from false-positive readings and background noise due to cell debris and medium contaminants. Specifically labeling MPs with Annexin V or other bio-labels can introduce artifacts as well, because not all MP types exhibit phosphatidylserine [12], [13], and we also could not find correlation between Annexin V to MP counts in a variety of vascular diseases [14], [15]. In addition, the binding of such bio-labels to MPs is influenced by the calcium concentration of the sample, and the membrane PS content [16]. Recently it was shown that fluorescent particles, possibly in the form of antibody aggregates, are present in commercial antibody solutions and these fluorescent particles can contribute to flow cytometry false-positive readings [17].

To better understand the nanostructure of MPs, direct imaging is a preferred characterization technique. However, due to their nanometric size, MPs are at the lower limit of resolution for conventional light microscopy techniques. Although confocal or florescence microscopy can detect the larger MPs (>200 nm), the fine details of these particles cannot be resolved [1]. Few attempts to use high-resolution microscopy have been published, but none seem quite successful, as the nanostructure of the MPs was somewhat altered by specimen preparation. Yuana et al. [18] implemented atomic force microscopy to characterize circulating MPs bound to mica via antibodies. Although they acquired measurements of the relative size distribution of MPs, fine features of these particles were not shown. Moreover, the MP surface binding treatment via antibodies may affect the morphology of MPs, thus hampering the determination of the real diameter. Other groups used TEM to visualise MPs. However the staining and drying procedures in preparing the specimens most probably altered much of the particles dimensions, and disrupted the fine morphology of the vesicles and the biomarkers linked to them [19]–[21]. Combes et al. [20] tried also SEM to image MPs and MP shedding from the cell surface. However due to their low SEM resolution, no actual nano-scale data was acquired.

Working with only fresh MP samples is highly labor-intensive, can complicate inter-institutional collaboration, and is clinically impossible. Therefore, in case a sample containing MPs is not dealt with or characterized a short while after its preparation, storage at sub-zero temperatures must be used to avoid alteration of the MPs nanostructure due to probable lipid oxidation or proteolysis. Normally MP samples are frozen and stored at –80°C. However there is no standard and uniform protocol how to perform that properly [1]. As different storage methods may alter the results obtained from the same sample, developing an adequate and standard procedure for storing MPs by freezing is important to current research, and will be even more critical in the future, if these particles are used as a diagnostic tool. A number of publications discuss the influence of freezing MP samples for storage. Those who checked the influence of freezing on MPs did so by relying only on flow cytometry (particle counts and/or specific antigen expression). Some concluded that there is no difference between fresh samples and those frozen to –80°C, and thawed only once [12], [22]–[24]. Others showed that if samples were "snap-frozen" in liquid nitrogen (LN_2_) prior to storage in –80°C, there were no major differences compared to fresh samples [25], and if samples were thawed to RT or 37°C in a heated water bath (not ice) after their freezing, the results were even better [26]. Conversely, others have reported that there was a significant decrease in the numbers of MPs after a freeze-thaw cycle [27], and other publications suggested that there was an increase in MP numbers after a freeze-thaw cycle [28]. Obviously there are major contradictions between the results presented in the literature concerning how freeze-thaw cycles affect the behavior of the MP samples. These differences may be possibly explained by the use of MPs of different origins and stimuli, but it also should be kept in mind that flow cytometry is an indirect method with limited resolution; it also depends on how the readings are interpreted, and it does not truly show how the nanostructure of the particles change by the different treatments. Thus, high-resolution direct-imaging could shed light on this important issue.

Cryogenic transmitting electron microscopy (cryo-TEM) is used for high-resolution imaging of high vapor-pressure specimens. These samples are aqueous or non-aqueous systems, such as suspensions, emulsions, or biological specimens, and even solids of high vapor pressure. One needs cryogenic conditions to avoid loss of volatiles from the specimen in the high vacuum of the TEM. Sufficiently low temperatures reduce the vapor pressure to negligible levels. Those conditions also arrest motion on the supra-molecular level, which could lead to blurry images. Fast enough cooling vitrifies the specimen, thus preserving its nanostructure. Cryo-TEM provides high-resolution direct images of domain sizes, from a few to about 300 nanometers, and elucidates the nature of the basic building blocks of the studied system. As such systems often include different coexisting assembly types, cryo-EM allows observing them all. Data interpretation from cryo-TEM is often straightforward, not model-dependent [29]–[33].

As thermal fixation by rapid cooling, rather than chemical fixation, is a preferable technique for preparing labile specimens, while retaining their native nano-structure [34], the thin-film vitrification method pioneered by Dubochet and his colleagues [35] has been proven successful in preparing specimens for cryo-TEM. This methodology has been successfully applied to characterize cryo-TEM vitrified specimens of a wide range of complex liquids for more than 25 years [36]–[38]. While cryo-TEM has become an important direct-imaging method, one must take into account that it cannot be used for highly viscous samples, or those containing large objects, because the specimens must be very thin (100–300 nm thick), and larger objects are excluded from the specimen during preparation [29]. Thicker specimens or those containing large object can be imaged by cryo-electron tomography [39], or by cryo-SEM [40].

## Methods and Materials

### Collection and isolation of microparticle samples

**Plasma Microparticles (PMPs).** Venous blood was drawn from healthy volunteers into 3.2% sodium citrate tubes. All volunteers provided written informed consent and the study was approved by the Institutional Review Boards (IRBs) of the Rambam Health Care Campus (Approval # 2030). Within one hour of collection, blood was centrifuged twice at 1500 g for 15 minutes to separate cells and platelets from blood plasma. The poor platelet plasma (PPP) supernatant was divided into 2 mL aliquots centrifuged at 18,000 g for one hour. MP-free supernatant was removed, and the MP pellet was resuspended in 100 µL of phosphate-buffered saline (PBS) [15], [41]. Storing conditions were divided to two groups:

- **Fresh PMPs:** Samples were stored at 4°C, and were used within 24 hours.
- **Frozen PMPs:** Samples were frozen at –80°C, and after up to a week were passively thawed to room temperature (RT), up to 30 minutes before use.

**THP-1-derived MPs.** THP-1 human acute monocytic leukemia cell line cells were cultured in medium consisting of RPMI-1640 medium supplemented with 10% fetal calf serum (FCS), 1% antibiotics (10 mg/mL streptomycin). Cells were incubated in Nunclone plates or flasks at 37°C, 5% CO_2_. MPs were collected according to Aharon et al. [42] with some alterations. After reaching cell concentration of about 5×10^5^ cell/mL, cells were stimulated to microvesiculate by 10 µM calcium ionophore A23187 (Sigma-Aldrich Israel Ltd., Rehovot, Israel) for 20 minutes [43]. The MPs were isolated from the cells by multiple centrifugations: 1,500 g for 10 min twice, supernatant was collected. MPs suspensions were spun down twice at 18,000 g for one hour, and each time washed with PBS to a final volume of 100 µL (modified protocol from Jaiswal et al. [44]). Storing conditions were divided into four groups:

- **Fresh THP-1 MPs:** Samples were stored at 4°C, and were used within 24 hours.
- **Frozen THP-1 MPs:** Samples were frozen at –80°C, and were passively thawed to room temperature up to 30 minutes before use.
- **Frozen THP-1 MPs+10% DMSO:** Before freezing, 10% of dimethyl sulfoxide (DMSO) was added to each vial. Samples were frozen at –80°C, and passively thawed to room temperature (RT) up to 30 minutes before use.
- **Rapid-cooled THP-1 MPs:** Samples were rapid-cooled to –196°C in LN_2_, and actively thawed, using a heated water bath, to 37°C up to 30 minutes before use.

All frozen samples were thawed after up to a week from initial freezing.

**MDA231-derived MPs.** MDA231 estrogen receptor negative, epithelial breast cancer cell line cells were cultured in medium consisting of Dulbecco's Modified Eagle Medium (DMEM) supplemented with 10% FCS, 1% antibiotics (10 mg/mL streptomycin). Cells were incubated in Nunclone flasks at 37°C, 5% CO_2_. After reaching a cell count of about 2×10^7^ cells, cell blebbing was induced by a 24–48 hours starvation period (cells were cultured in medium without serum as adapted from a protocol of Hoyer et al. [45]). The cell medium, rich in MPs, was collected and centrifuged twice at 1500 g for 10 minutes to precipitate unwanted cell fragments. The supernatant was collected, and the MPs were separated using three different methods:

- **Centrifugation**: First centrifugation at 18,000 g for an hour [44]. To increase the MP concentration threefold, three samples were combined, and then centrifuged again at 18,000 g for an hour, and finally suspended in 100 µL of PBS.
- **Filtration**: The supernatant was filtered through a 0.22 µm PVDF syringe filter (Millipore Ireland Ltd., Cork, Ireland). Particles, such as MPs that did not pass through the filter, were back-washed with PBS into aliquots of 100 µL.
- **Dialysis**: One liter of dialysis solution made of 20% wt. 20 kDa poly-ethylene-glycol (PEG; Merck Schuchardt OHG, Hohenbrunn, Germany) in PBS was prepared. 10 mL of the MP solution was inserted into a dialysis tube with a cut-off of 12–14 kDa (Spectrum Laboratories Inc., Rancho Dominguez, CA, USA). The tube was immersed in the dialysis solution at 4 °C. The dialysis ended after 3.5 hours, when only about 200 µL of the original solution remained in the tube. The solution was split into 4 portions of 50 µL each.

All MDA231 MP samples were freshly used or rapid-cool stored as described above.

**HVT-derived MPs.** Human villous trophoblasts obtained at 20–24 weeks of gestation were purchased from ScienCell (Carlsbad, CA, USA). Cells were cultured in a modified culture medium, consisting of 50% trophobalst medium with supplements, 22% DMEM, 22% F12, 4% fetal calf serum (FCS), 1% antibiotics (10,000 units/mL penicillin, 10 mg/mL streptomycin, 250 units/mL nyastatin), 0.0001% amphotericin B. Cell blebbing was induced by 48 hours starvation (cells were cultured in medium without serum; after Hoyer et al. [45]). The cell medium was collected, and centrifuged twice at 1500 g for 10 minutes. The MP supernatant was collected, and centrifuged once at 18,000 g. The supernatant was removed, the MP pellet was suspended in 100 µL of PBS, and stored using the rapid-cooling technique. To ensure that the vesicles in the medium are indeed MPs, not membranous blebs or remnants of the plasma membrane of dying cells, FACS analysis was used. HVT cells were washed and labeled with Calcein AM Fluorescent Dye (BD Bioscience, San Jose, CA, USA), which converted to a green-fluorescent Calcein after cetoxymethyl ester hydrolysis by intracellular esterases. Then, cells were starved for 48 hours, and MPs were isolated from the cell supernatant by centrifugation (18,000 g, 1 hour). MP pellet was screened by a CyAn ADP flow cytometer (Beckman Coulter, Nyon, Switzerland) using the 488 nm (FL1) solid-state laser, and compared to 0.78 µm green fluorescent size calibration beads and to unlabeled HVT-MPs. Only MPs that create complete vesicles would exhibit green fluorescent cytoplasm, which can be examined by FACS, while membrane fragments would stay unlabeled.

### Cryogenic transmission electron microscopy (Cryo-TEM)

Specimen preparation took place in a home-made controlled environment vitrification system (CEVS) [30]. Specimens were prepared at a constant temperature of 25 or 37°C. To prevent solvent evaporation and changes in solvent concentration, the specimens were prepared in a chamber at 100% relative humidity [31]. Before sample preparation, the sample grids were made more hydrophilic using a PELCO EasiGlow glow-discharge apparatus (Ted Pella Inc., Redding, CA, USA). A drop of the sample was pipetted onto a carbon-coated perforated polymer film, supported by a 200 mesh TEM grid (Ted Pella Inc., Redding, CA, USA) held by tweezers inside the chamber. The drop was thinned into a film less than 300 nm thick, by blotting away excess solution with a filter paper wrapped on a metal strip. The grid was then plunged (dropped mechanically) into liquid ethane at its freezing point (–183°C) cooled by LN_2_ at its boiling point (–196°C). Until imaging, the vitrified specimens were stored under LN_2_.

Cryo-specimens were examined in a Philips CM120 or an FEI T12 G^2^ cryo-dedicated transmission electron microscopes (Eindhoven, The Netherlands), operated at 120 kV, using either an Oxford CT-3500 (for the CM120 microscope; Oxford Instruments, Abingdon, England), or a Gatan 626 (for the T12; Gatan, Pleasanton, CA) cooling holders and transfer stations. Specimens were equilibrated in the microscopes below –178°C, examined in the low-dose imaging mode (no more than 20 electrons per Å^2^) to minimize electron beam radiation damage, and recorded at a nominal underfocus of about 2 µm to enhance phase-contrast. Images were acquired digitally by a MultiScan 791 (CM120) or a US1000 (T12) cooled charge-coupled-device cameras (Gatan, Pleasanton, CA), using the Digital Micrograph software (Gatan UK, Abingdon, UK).

### Room temperature transmission electron microscopy (RT-TEM)

Some MDA231-derived MP samples, separated by centrifugation, were imaged by RT-TEM. The samples were prepared by uranyl acetate negative-staining as described by Von der Malsburg et al. [46]. This technique involves the preparation of a 2% aqueous solution of uranyl acetate using uranyl acetate dehydrate powder (Merck, KGaA, Darmstadt, Germany) dissolved in DDW. To adsorb the sample on its carrier, a 400-mesh carbon-coated grid was placed on a 20 µL sample drop for 2 min at RT. Excess sample was removed with a filter paper. The sample was rinsed by swiping the grid on a drop of PBS, excess solution was blotted. To negatively stain the sample, the grid was placed, film side down, on a fresh 20 µL drop of 2% uranyl acetate for 2 min, followed by blotting and air drying. TEM imaging was performed at RT using the Tecnai T12 G^2^ electron microscope, using its room-temperature specimen holder. Images were acquired using the same camera and software as done with cryo-TEM.

## Results

### Circulating microparticles

**Figure 1:**
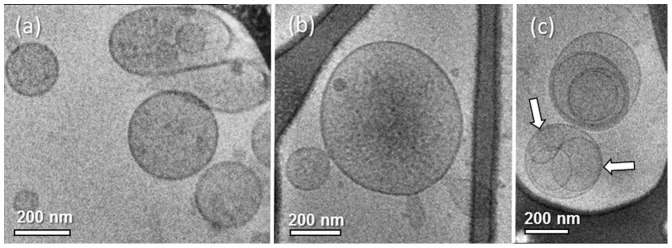
Cryo-TEM images of circulating MPs withdrawn from the blood of a healthy subject. (a) MPs are seen to be flexible as they can be compressed between two surfaces; (b) shedding of smaller vesicles from an MP; (c) smaller vesicles incapsulated inside bigger MPs, creating multilayered MPs. Inner vesicles deform according to the outer MP membrane shape (arrows).

We collected and separated MPs withdrawn from the blood of a healthy subject, and imaged them using cryo-TEM. Most of these particles were spherical with dimensions of 200–400 nm. Some of the MPs seemed to have flexible membranes, as seen by their ability to be compressed between the two water-air surfaces and the walls of the holes in the carbon film (Fig. 1a). In other occasions we saw that smaller vesicles shed from the surface of bigger MPs (Fig. 1b). We also detected MPs with smaller vesicles residing in them (Fig. 1c). We termed these formations "multilayered MPs". It is possible to determine that the outer MP truly encapsulates the inner vesicles, as the morphology of the inner ones deform according to the membrane of the outer MP (arrows).

**Figure 2:**
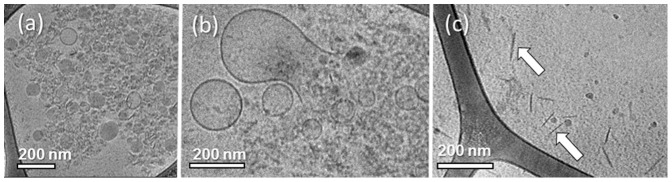
Cryo-TEM images of circulating MPs after storage at–80°C prior to specimen preparation. (a) MP inner contents are filling the sample medium; (b) MP breakage is documented; (c) pin-shaped nanoparticles, possibly membrane fragments seen side-on (arrows).

We repeated the experiment with samples that had been stored at –80°C, and thawed at RT prior to cryo-TEM specimen preparation. In that trial the solvent was full of particles, which we saw filling the MPs in the fresh samples (Fig. 2a). In Figure 2b we see that MP membrane breakage is the reason why the MP contents spilled-out into the sample medium. We also noticed many pin-shaped nanoparticles that we believe to be membrane fragments positioned perpendicular to the e-beam (arrows in Fig. 2c). Based on these data we believe that as a result of the slow freezing, ice crystals grow and puncture the MP membranes. The slow freezing also increases considerably the osmotic pressure within the sample volume that can rapture the soft nanostructures. This increase in osmotic pressure happens due to the elevated concentration of solutes expelled from the developing ice crystals to the residual unfrozen water between ice crystals. Eventually, during the sample thawing, the MPs burst.

### THP-1-derived MPs

**Figure 3:**
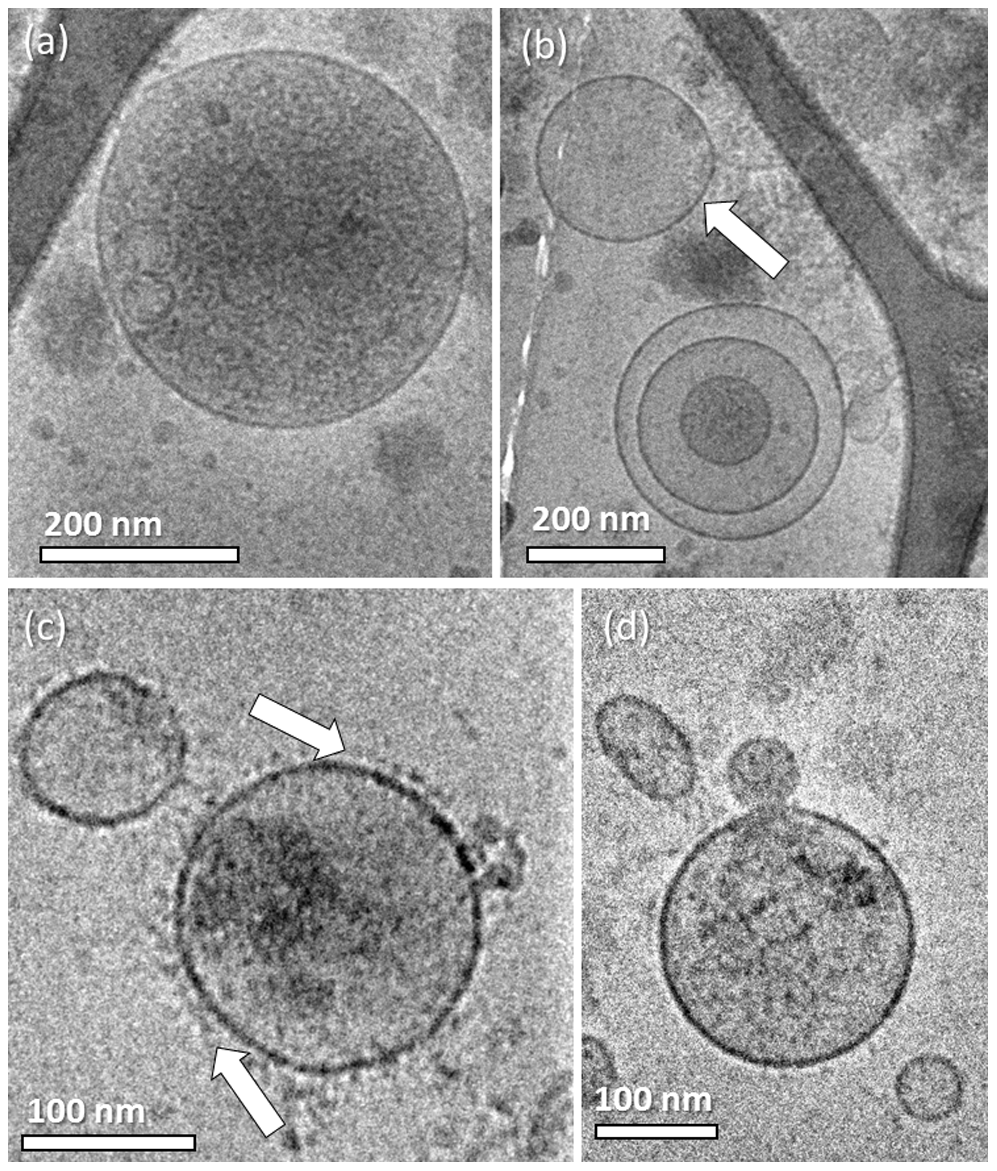
Cryo-TEM images of THP-1 derived MPs. (a) Heavily granulated MP; (b) multilayered MP with two inner vesicles; arrow indicates a very smooth, almost empty MP; (c) arrows show nanoparticles, probably glycoproteins, decorating the MP membrane; (d) vesicle shedding from the MP membrane.

To thoroughly investigate if the freeze-thaw cycle induced artifacts, we chose to work with a system that would make it easier to acquire MPs rather than searching for candidates to withdraw blood from. Therefore we worked with the THP-1 human acute monocytic leukemia cell line. We used 10 µM calcium ionophore A23187 to induce cell stimulation. The THP-1 derived MPs appeared mostly spherical, with dimensions from 200 to 400 nm. We were able to locate some MPs that were heavily granulated, and full of granular content, which probably came from their parent cells (Fig. 3a), while others were very smooth, having low concentration of particles in them (arrow in Fig. 3b). On some MPs, we could clearly see a nanoparticle corona surrounding them. These nanoparticles seemed embedded in the membrane, probably glycoproteins that originate from the surface of the parent cell (arrows in Fig. 3c). We also noticed multilayered MPs (Fig. 3b), and vesicle shedding (Fig. 3d), similarly to what we had seen in the circulating MPs system.

**Figure 4:**
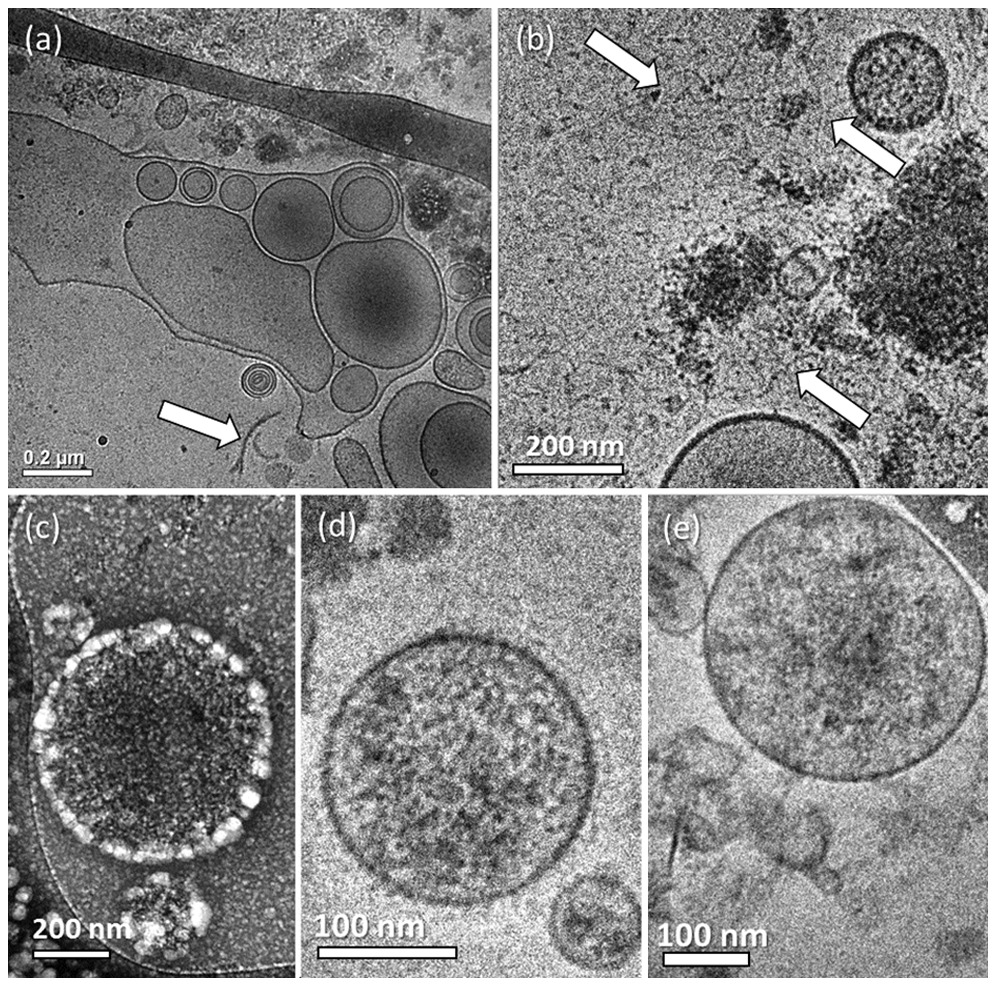
Cryo-TEM images of THP-1 derived MPs after freezing to–80°C. (a) Sample freezing induced numorous artifacts such as the fomration of massive membrane structures enclosing smaller MPs and membrane breakage (arrow); (b) elongated nano-fibers are seen, possibly DNA or RNA strands explled from the broken MPs; (c) sample with 10% v/v DMSO; although the MP morpholgy seems intact, severe e-beam radiation damage is seen; (d-e) samples that were rapid-cooled using LN_2_ before storage at -80°C; MPs showed the same morphology seen in the fresh samples.

We repeated the experiment with samples stored at –80°C prior to specimen preparation. As expected, many morphological artifacts were seen due to the sample freezing. In Figure 4a, one can see that large membranes enclosing smaller MPs; membrane fragments were also seen (arrow). These formations can obviously alter flow cytometry analysis and obscure readings of the inner vesicles. In certain images we detected elongated nano-fibers (arrows in Fig. 4b). We believe that these nanostructures are DNA or RNA strands expelled to the sample medium after their hosting MPs burst open. We base our suggestion on the similarity of these nanofibers to DNA ejected from disintegrating lipoplexes in serum, as imaged by cryo-TEM [47].

To slow down the development of ice crystals that result in punctured MP membranes, we tried to use dimethyl sulfoxide (DMSO), an efficient cryoprotectant, commonly used in cryogenic cell preservation [48]. We decided to work at a concentration of 10% v/v, considered standard [49]. It is possible that the cryoprotectant preserved the morphology of the MPs during the freeze-thaw cycle. However, as seen by the numerous holes between the MP membrane and solvent, the specimen had become extremely sensitive to electron beam radiation damage, even when low-dose imaging was practiced (Fig. 4c). This sensitivity can be explained by the high concentration of electrons in the DMSO molecule, resulting in increased production of free-radicals by the e-beam. The radiation damage artifacts did not allow acquiring images with high enough resolution, thus we chose to work with a different technique. We decided to minimize the ice-crystal production and the effects of elevated osmotic pressure by rapid-cooling and quickly thawing the sample. To do so, we immersed the test-tube containing the specimen in LN_2,_ and only than it was stored at –80°C. The thawing was done in a water-heated bath at 37°C. This preservation technique led to a minimum number of distorted MPs; much less MP debris and expelled contents were noticed in the medium, and eventually most of the MPs seemed as normal as seen in the fresh samples (Figs. 4d&e). Because of these good results, all the following MP samples were prepared fresh, or rapid-cooled by LN_2_ prior their storage at –80°C.

### MDA231-derived MPs

**Figure 5:**
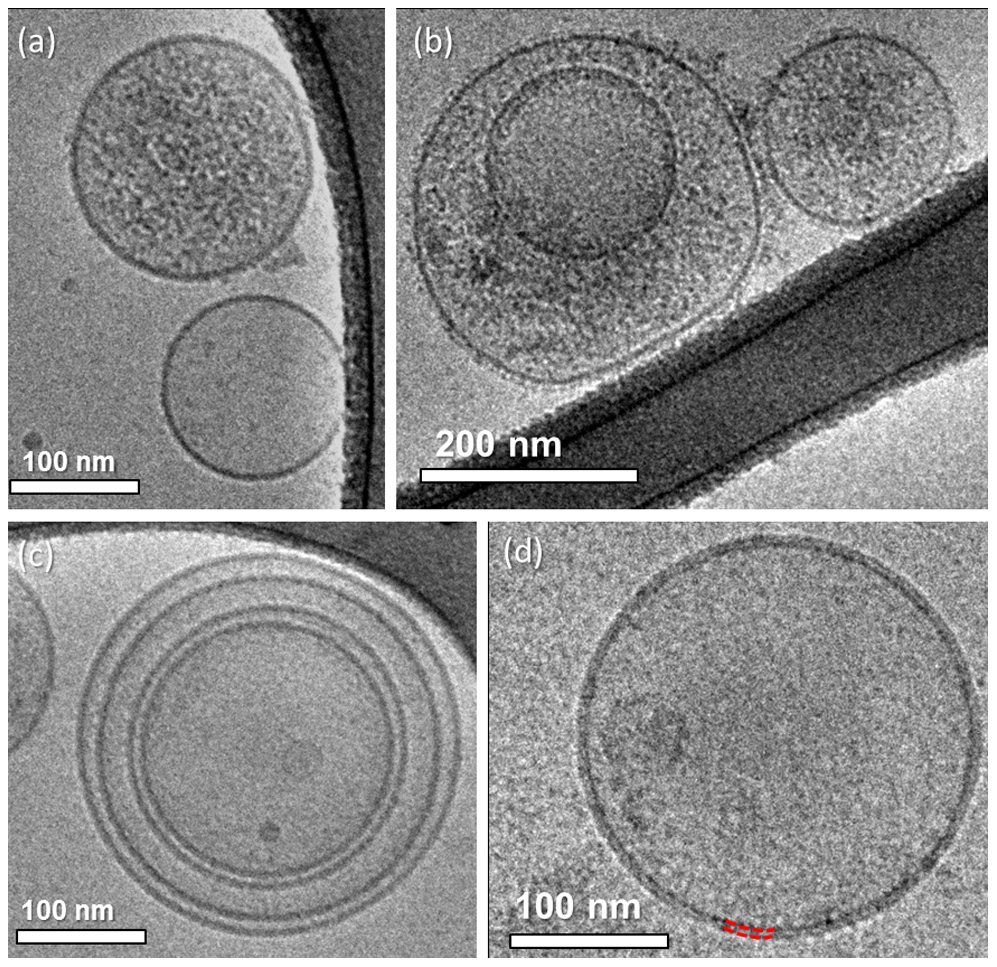
Cryo-TEM images of MDA-231 derived MPs. (a) Granulated and smooth MPs; (b) bilayered MP with nanoparticle decoration on its membrane; (c) multilayered MP with three inner vesicles; (d) two dashed lines clearly show the phospholipid bilayer stucture of the MP membrane.

We studied the MPs derived from the estrogen receptor negative, epithelial breast cancer cell line MDA-231. To produce large amounts of MPs, cells were stimulated by starvation for 24–48 hours. As seen with above mentioned cell-derived MPs we studied, these particles were spherical, mostly having diameters of 150–400 nm (Fig. 5). We also noticed morphologies already seen in other MPs such as granulated versus smooth MPs (Fig. 5a), the decoration by nanoparticles attached to the MP membrane (Fig. 5b), and bilayered and multilayered MPs (Figs 5b&c). By working closer to focus, we resolved the phospholipid bilayer (dashed markings in Fig. 5d), and proved for the first time using direct imaging that these particles are truly vesicles.

**Figure 6:**
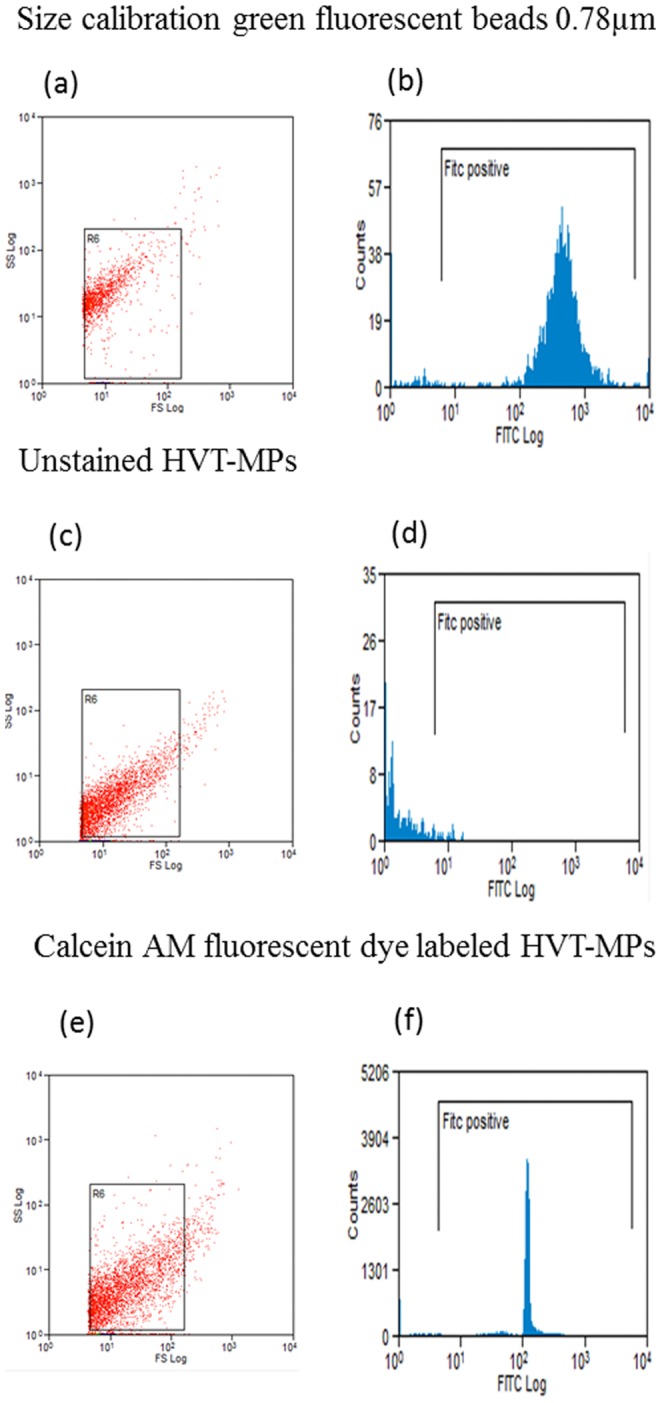
FACS analysis of HVT cell-derived MPs. (a) MP size evaluation gate (R1) established using 0.78 µm beads with side scatter (SS) and forward-scatter (FSC) in logarithmic scale; (b) fluorescence intensity of green beads (FITC); (c) non-labeled HVT-MP population; (d) non-labeled HVT-MPs at gate R1 show no fluorescence intensity (negative control); (e) HVT-MPs green labeled by Calcein AM fluorescent dye at gate R1; (f) fluorescence intensity–89% of MPs at gate R1 were green labeled.

We also investigated the morphology of MPs derived from human villous trophoblast (HVT) cells stimulated by starvation of 48 hours. As described above, we ensured that the vesicles in the medium were indeed MPs by labeling with Calcein AM. We induced MP shedding by starvation. FACS analysis demonstrated that 89% of MPs at gate R1-equivalent to 0.78 µm size beads were green labeled, indicating them to be true vesicles and not membrane fragments (Fig. 6)

**Figure 7:**
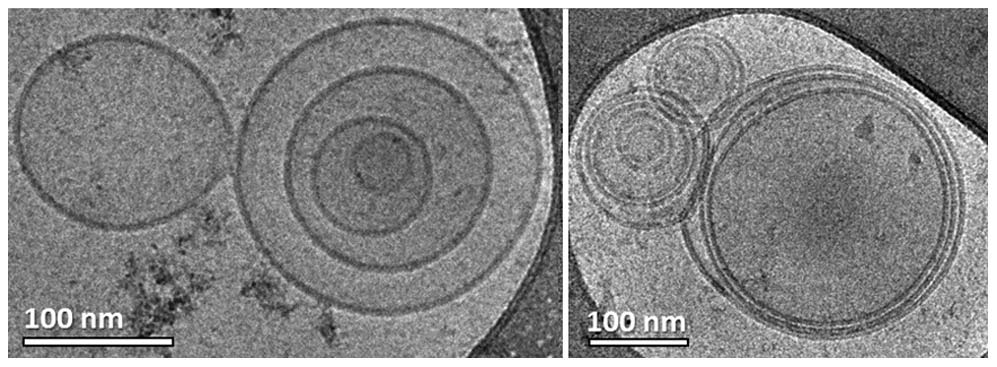
Cryo-TEM images of HVT cell-derived MPs; most of MPs were multilayered.

**Figure 8:**
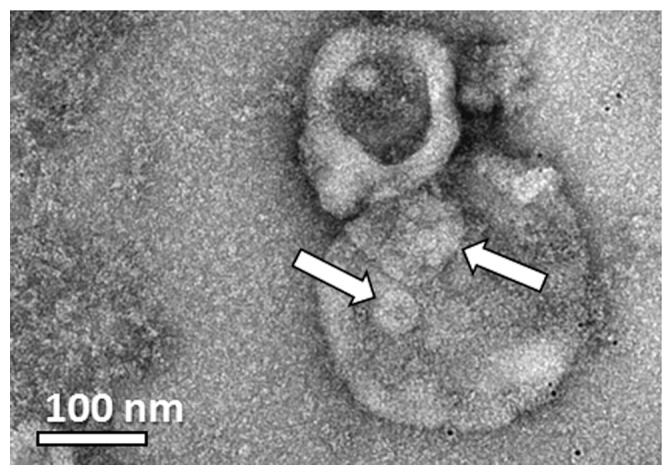
TEM image of a uranyl acetate negative-stained sample of MDA231 MPs: Multilayered MP morpholgy is seen. Arrows indicate vesicles seen in the main MP.

In addition we were surprised to see that almost all the HVT-derived MPs were multilayered (Fig. 7). This came as a surprise, because the theory of the mechanism of the MP shedding suggests only the production of monolayered MPs. Moreover, there is no evidence in the literature for this type of morphology. As we saw the multilayered morphology in all MP systems, we thought that the specimen preparation process could have been responsible for causing the multilayered morphology artifacts. From our experience it is known that blotting during cryo-TEM specimen preparation introduces high shear forces, which may alter the nano-structure of the sample [50]. Although we had not previously encountered a case in which multilayered vesicles were formed by sample blotting, it was reasonable to assume that the shear forces could break the vesicles, which then close back into multilayered formations. When we prepared specimens of MDA231 MPs using the uranyl acetate negative staining technique that did not involve blotting, RT-TEM imaging showed MPs with multilayered morphology as well (arrows in Fig. 8). Furthermore, from a kinetic point of view, as no intermediate formations were seen, it was not reasonable that all the MPs break, and then uniformly re-form multilayered ones in less than one second it took to thermally fix them after blotting.

**Figure 9:**
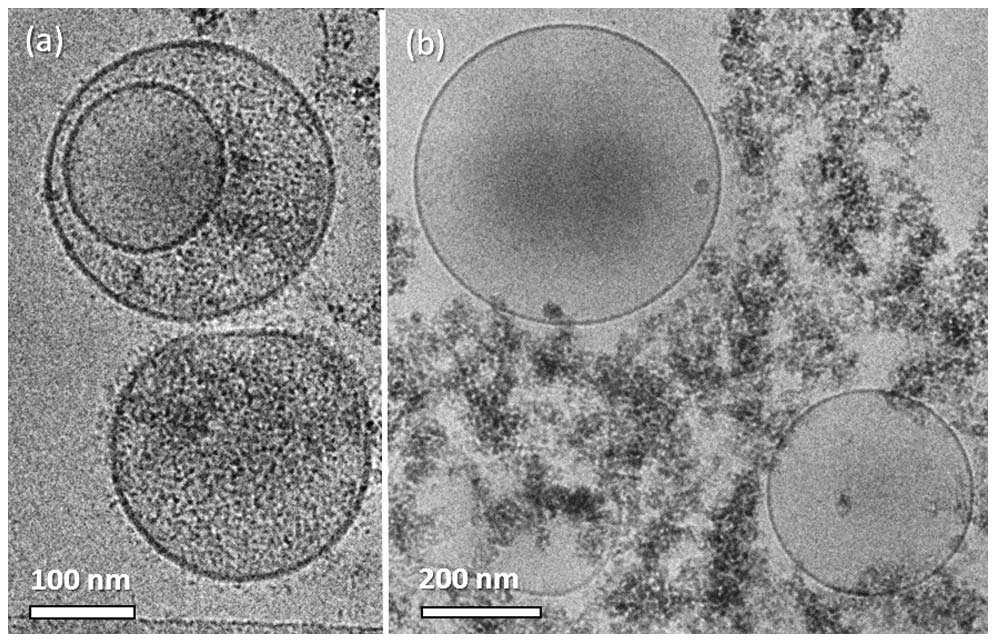
Cryo-TEM images of MDA231 MPs separated by filtration: (a) Only a few MPs showed a bilayered morphology; (b) contamination is dominating the sample, probably medium proteins that did not wash out.

Later on we suspected that the centrifugation of the samples at 18,000 g (for two hours in total) could force the smaller MPs into bigger ones, resulting in multi-layered formations. To check whether the separation technique created these artifacts, we decided to separate the MPs by the filtration of the MDA231 cell medium after the cells had been stimulated. By that technique we avoided the effect of continuous centrifugal forces on the particles, but did add shear forces that had to be taken into account. We used a 0.22 µm PVDF membrane to filter out all the smaller particles such as medium proteins, and retained bigger particles such as the MPs. Then we back-washed the membrane using PBS to form the samples. We were satisfied that by using this technique we were able to separate MPs that showed the same spherical morphology and size distribution as the ones separated by centrifugation (Fig. 9). We were pleased also to see that out of more than 100 MPs documented there were only two multilayered ones, and less than five bilayered ones (i.e. MPs that have only one vesicle in them), as seen in Figure 9a. Unfortunately the sample was contaminated by agglomerated nanoparticles (Fig. 9b) probably medium proteins that adhered to the membrane during filtration, and were added to the sample during the back-wash.

**Figure 10:**
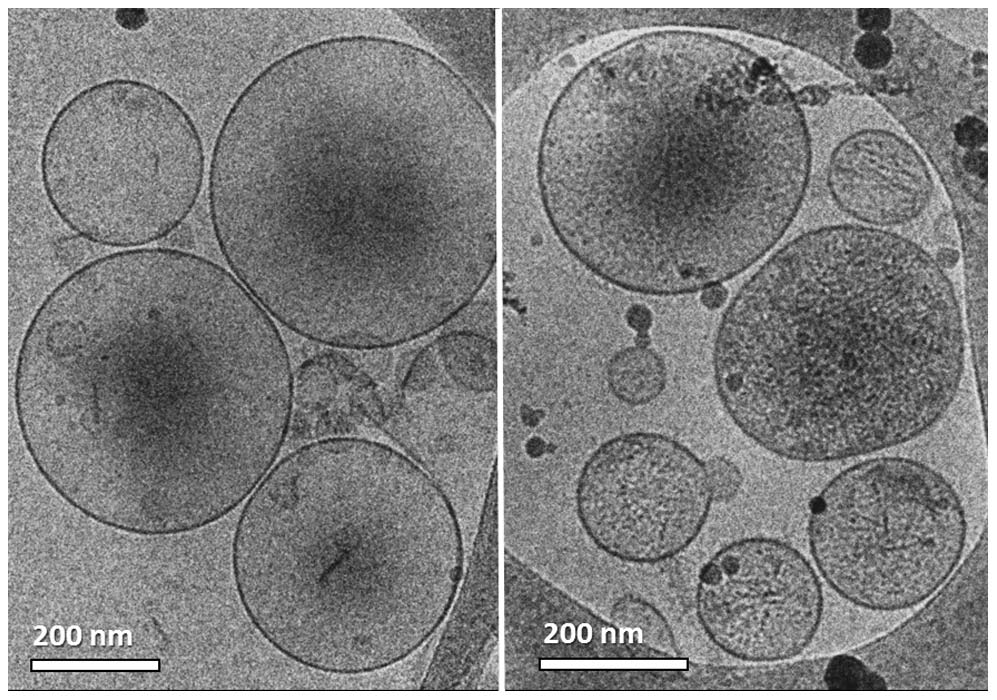
Cryo-TEM images of MDA231 MPs separated by dialysis: All MPs were spherical; no major contamination was seen.

As filtration can in principle break the MPs, dry them at the stage prior to the back-wash, and as it did not seem to clean the samples well enough, we chose to try separate the MPs by dialysis, a technique that to our knowledge, had not been used before. To concentrate the sample we immersed the dialysis tube containing the cell medium in a dialysis solvent of 20% wt. PEG in PBS. Due to osmosis the cell medium would cross the membrane (taking with it the smaller medium proteins), leaving the bigger MPs inside. By this method we concentrated the sample 50-fold in 3.5 hours at 4 °C. Because the samples became too viscous, making cryo-TEM specimen preparation impossible, we had to dilute them 5 times by PBS. We were able to locate many MPs of standard morphology after using this technique. It is important to mention that unlike other specimens we imaged, almost all the MPs were perfect spheres (Fig. 10). The specimen was very clean, especially in contrast to samples that had undergone filtration. We were very satisfied to image no multilayered MP, except for two bilayered ones out of more than 100 MPs documented.

## Discussion

High-resolution, direct imaging cryo-TEM was used to investigate the near-to-native nano-morphology of four different MP systems, and show that there are no major differences between MPs derived from different cells stimulated by several methods. We showed that most MPs are spherical having diameters from 150 to 400 nm (although bigger ones could have been excluded due to cryo-specimen preparation), and proved that they are truly vesicles by revealing their phospholipid bilayer membrane structure. However, it seems that each MP population was very heterogeneous, showing diverse morphologies, such as rough MPs enclosing high concentrations of dispersed cargo, as opposed to smoother ones, which seemed rather empty, and vesicles that were decorated by many nano-particles protruding from their membrane. The subgroups of MPs we imaged may imply the diverse multiple biological roles associated with MPs.

Unlike the contradicting results shown in earlier reports by flow cytometry, we showed by direct imaging that the most common method used to store MPs, namely, slow freezing to –80°C, may lead to many morphological artifacts. To minimize the artifacts caused by the generation of ice-crystals and elevated osmotic pressure during freezing, we tried to use DMSO as a cryoprotectant. Although it seemed that most MPs retained their original nanostructure after a freeze-thaw cycle, the specimen became too sensitive to e-beam radiation, making it impossible to acquire acceptable enough images. As Trummer et al. [26] showed by flow cytometry, to overcome this problem we rapid-cooled the samples in LN_2_ prior storage at –80°C, and quickly thawed the sample to 37°C using a heated water bath. This method gave similar results to those seen in fresh samples, and minimized the artifacts seen when the samples were slowly frozen. Thus, our results show that the current method for storing MPs must be further investigated to minimize unwanted artifacts. Standardising the storing method to reduce the formation of artifacts is important because MPs are studied by many research groups, and are considered to become a means of a diagnostic tool in the future.

In all investigated MP systems we noticed that a portion of the particles showed a multilayered morphology. While some MPs had only one vesicle in them (termed by us 'bilayered MPs'), others had more, and sometimes several smaller multilayered vesicles in one MP. As that morphology was not supported by the theory of MP self-assembly, we tested whether those structures were artifacts. After we concluded that cryo-TEM specimen preparation technique could not cause the formation of multilayered structures, we checked whether the MP separation method by centrifugation (at 18,000 g for two hours) could push one MP into another. By using novel MP separation techniques of filtration or dialysis, we saw a dramatic decrease in bilayered and multilayered morphologies, a result that suggests that those structures are artifacts caused by the centrifugation. From these preliminary results we deduce that there is a strong effect of the separation technique on the MP observed morphology. We can see that by using centrifugation there is an increased number of multilayered MPs. Although we need to run more tests to verify our results, one must take into account that the current, most common method to separate MPs may introduce artifacts to the system.

Based on these results, we are convinced that a multidisciplinary study combining work methodologies and viewpoints of both life and soft material sciences could contribute to the on-going research of these fascinating nano-particles, by standardizing current working procedures and creating a link between the MP nano-morphology to its biological function. The question whether the methodology described here could be used routinely as a diagnostic tool cannot be answered yet, as it depends on the degree of sophistication a hospital clinical TEM laboratory is willing to reach.
